# Clinical characteristics and outcomes of 144 critically ill children receiving therapeutic plasma exchange in a pediatric intensive care unit: a single-center retrospective study

**DOI:** 10.3389/fped.2026.1955886

**Published:** 2026-10-16

**Authors:** Zhijiang Chen, Qiuju Wei, Jufang Liu, Cheng Zeng, Wei Zhao

**Affiliations:** Pediatric Intensive Care Unit, Pediatric Center, Zhujiang Hospital, Southern Medical University, Guangzhou, Guangdong, China

**Keywords:** apheresis, critically ill children, multiple organ dysfunction syndrome, pediatric intensive care unit, prognosis, therapeutic plasma exchange

## Abstract

**Background:**

Therapeutic plasma exchange (TPE) is increasingly used in critically ill children, yet pediatric data remain scarce. This study aimed to characterize the clinical features, indications, complications, and outcomes of children receiving TPE in a pediatric intensive care unit (PICU).

**Methods:**

We retrospectively enrolled 144 children who underwent TPE in the PICU of Zhujiang Hospital, Southern Medical University, between January 2017 and December 2021. Demographic data, diagnoses, TPE indications (categorized according to the 2019 American Society for Apheresis [ASFA] guidelines), complications, and outcomes were collected. Multivariate logistic regression was performed to identify risk factors for mortality.

**Results:**

The 144 children (median age, 66 months; median Pediatric Risk of Mortality III score, 18) underwent 414 TPE sessions. The leading indications were neurological disorders (29.2%), liver diseases (19.4%), and hematologic disorders (16.7%); 57.6% of patients were treated for ASFA category III indications. The complication rate was 16.9% per session, with filter or circuit clotting the most frequent event (7.2%); all complications resolved after prompt management, and no treatment-related death occurred. Overall PICU mortality was 36.8%, highest in the liver disease group (60.7%) and lowest in the neurological disorder group (16.7%) (*P* = 0.012). Non-survivors had higher rates of multiple organ dysfunction syndrome (MODS), mechanical ventilation, and vasopressor use than survivors (all *P* < 0.001). Multivariate analysis identified MODS (odds ratio [OR] = 5.609, 95% confidence interval [CI]: 1.189-26.453) and vasopressor use (OR = 6.551, 95% CI: 1.310-32.768) as independent risk factors for mortality.

**Conclusions:**

TPE is generally safe in critically ill children when performed in an experienced PICU. MODS and vasopressor requirement independently predicted death. Because no control group was included, treatment efficacy could not be evaluated; our conclusions are limited to the safety and clinical profile of TPE in this population.

## 1 Introduction

Therapeutic plasma exchange (TPE) is an extracorporeal blood purification technique that separates plasma from blood cells, removes pathogenic macromolecular substances (such as autoantibodies, immune complexes, cryoglobulins, endotoxins, and cytokines), and replaces the removed plasma with albumin, fresh frozen plasma, or a crystalloid-colloid combination, thereby restoring internal homeostasis. It is particularly suitable for eliminating substances with a molecular weight exceeding 15,000 Da and a long half-life (1, 2).

TPE has been widely applied in the management of sepsis with multiple organ failure, hemolytic uremic syndrome, acute liver failure, neurological disorders (e.g., Guillain-Barré syndrome, myasthenia gravis, and autoimmune encephalitis), and drug or toxin poisoning (1–3). In 2019, the American Society for Apheresis (ASFA) published the eighth special issue of its evidence-based guidelines, which classifies indications for therapeutic apheresis into four categories and serves as the principal framework for treatment decision-making in both adults and children (4); the guidelines were further updated in the ninth special issue in 2023 (5).

Although the number of diseases amenable to TPE in the PICU continues to grow, pediatric clinical evidence remains limited: most published data derive from case reports or small retrospective series, and large-scale pediatric studies are rare (3, 6, 7). Moreover, pediatric TPE faces multiple challenges, including difficult vascular access, a high ratio of extracorporeal circuit volume to total blood volume (which predisposes to hypovolemia), treatment protocols (dose, frequency, and course) largely extrapolated from adult studies, and limited availability of equipment and trained personnel in primary-level institutions (1–3, 6, 7).

We therefore conducted a retrospective analysis of critically ill children who received TPE in our PICU between January 2017 and December 2021, aiming to characterize clinical indications, complications, and outcomes, and to identify factors associated with prognosis, thereby contributing practice-based evidence to the scarce body of pediatric TPE data.

## 2 Methods

### 2.1 Study design and patients

We retrospectively reviewed the medical records of critically ill children who underwent TPE in the Pediatric Intensive Care Unit, Pediatric Center of our hospital between January 2017 and December 2021. The following data were extracted: demographic information at PICU admission (age, sex, body weight, and diagnosis); baseline characteristics and treatment-related variables, including the presence of multiple organ dysfunction syndrome (MODS), the Pediatric Risk of Mortality III (PRISM III) score, mechanical ventilation and its duration (days), vasopressor support and its duration (days), PICU and total hospital length of stay, and survival status; and TPE-related data, including indications, complications, number of sessions, concomitant continuous renal replacement therapy (CRRT), and time to TPE initiation. MODS was defined as dysfunction of two or more organs at PICU admission, in accordance with the International Pediatric Sepsis Consensus Conference definitions (8). Severity of illness was assessed using the PRISM III score (9). Time to TPE initiation was defined as the interval (hours) from PICU admission to the start of the first TPE session. The primary outcome was PICU mortality, defined as death from any cause during the PICU stay. Children who had potential indications for TPE during the same period but did not undergo the procedure were not systematically recorded, and the proportion of eligible patients who ultimately received TPE could not be determined.

### 2.2 TPE indications and ASFA classification

All patients were classified according to the 2019 ASFA guidelines (4) as follows: category I, diseases for which TPE is accepted as first-line therapy; category II, diseases for which TPE is accepted as second-line therapy, either alone or in combination with other treatments; category III, diseases for which the optimal role of TPE is not established and decision-making should be individualized; category IV, diseases for which published evidence demonstrates or suggests that TPE is ineffective or harmful; and not classified, for diagnoses not listed in the ASFA guidelines.

### 2.3 TPE procedure

Vascular access was established with a double-lumen hemodialysis catheter placed in the internal jugular, subclavian, or femoral vein, with the catheter size selected according to the child's body weight and age. All procedures were performed in single-volume membrane-filtration plasma exchange mode using one of the following blood purification devices, chosen according to machine availability at the time of the treatment decision: Prismaflex (Baxter, USA), multiFiltrate (Fresenius Medical Care, Germany), or Plasauto *Σ* (Asahi Kasei, Japan), with matched filters and tubing sets. Replacement fluids included albumin solution, fresh frozen plasma, or a combination, at the discretion of the attending physician, and anticoagulation was individualized. Each session consisted of the exchange of 1.0–1.5 times the estimated plasma volume, and sessions were performed daily or on alternate days according to the underlying disease and clinical response. Fresh frozen plasma (FFP) was the default replacement fluid; in selected patients, a combination of albumin and FFP was used to balance colloid support against the risk of transfusion-related reactions. Unfractionated heparin was the first-line anticoagulant, and regional citrate was used in patients at high risk of bleeding. When the extracorporeal circuit volume exceeded 10% of the child's total blood volume, the circuit was primed with albumin or packed red blood cells to prevent hypovolemia.

### 2.4 Statistical analysis

Data were analyzed using SPSS Statistics version 21.0 (IBM Corp., Armonk, NY, USA). Categorical variables are presented as frequencies (*n*) and percentages (%) and were compared using the chi-square test. Continuous variables are expressed as mean ± standard deviation when normally distributed and as median with the 25th and 75th percentiles (interquartile range) otherwise; the Shapiro–Wilk test was used to assess the distribution of variables. Between-group comparisons for non-normally distributed variables were performed using the Kruskal–Wallis test, and one-way analysis of variance was used for normally distributed variables; for two-group comparisons, the Student t test and the Mann–Whitney U test were applied as appropriate. A binary logistic regression model was used to calculate odds ratios (ORs) with 95% confidence intervals (CIs) for factors associated with mortality. A two-sided *P* value < 0.05 was considered statistically significant.

### 2.5 Ethics

This study was approved by the Ethics Committee of Zhujiang Hospital, Southern Medical University (approval number: 2026-KY-250-01). Given the retrospective design and the use of anonymized data, the requirement for individual informed consent was waived by the committee. The study was conducted in accordance with the Declaration of Helsinki.

## 3 Results

### 3.1 Clinical characteristics, TPE indications, and complications

The baseline characteristics and treatment profile of the 144 children are summarized in Table 1. A total of 414 TPE sessions were performed (mean, 2.89 sessions per patient). The distribution of indications is shown in Table 2: neurological disorders (29.2%), liver diseases (19.4%), and hematologic disorders (16.7%) were the three leading indication groups, and 57.6% of patients were treated for ASFA category III indications. The overall incidence of procedure-related complications was 16.9% per session (70/414); filter or circuit clotting was the most common complication (7.2%), followed by hypothermia (2.4%) (Table 3). All complications resolved after prompt management, every patient completed the planned treatment course, and no treatment-related death occurred.

**Table 1:** Clinical characteristics of the 144 children who received therapeutic plasma exchange.

Characteristic	Median (IQR) or *n* (%)
Sex (male/female), *n* (%)	83 (57.6)/61 (42.4)
Age (months)	66 (24, 120)
Body weight (kg)	18.0 (12.0, 30.7)
MODS, *n* (%)	64 (44.4)
Mechanical ventilation, *n* (%)	72 (50.0)
Duration of mechanical ventilation (days)	1 (0, 10)
Vasopressor use, *n* (%)	42 (29.2)
Duration of vasopressor support (days)	2 (1, 2)
Total TPE sessions, n	414
TPE sessions per patient, mean	2.89
PRISM III score	18 (14, 22)
PICU length of stay (days)	12 (7, 26)
Total hospital length of stay (days)	21 (10, 36)
PICU mortality, *n* (%)	53 (36.8)

Data are presented as median (interquartile range) unless otherwise indicated.

IQR, interquartile range; MODS, multiple organ dysfunction syndrome; PRISM III, Pediatric Risk of Mortality III; TPE, therapeutic plasma exchange; PICU, pediatric intensive care unit.

**Table 2:** TPE indications and ASFA categories.

Clinical diagnosis	Patients, *n* (%)	TPE sessions, n	ASFA category
Sepsis	19 (13.2)	43	III
Liver diseases	28 (19.4)	119	
Acute liver failure	28	119	III
Renal diseases	10 (6.9)	29	
Hemolytic uremic syndrome	10	29	III
Hematologic disorders	24 (16.7)	65	
Graft-versus-host disease	5		Not classified
Hemophagocytic lymphohistiocytosis	11		III
Macrophage activation syndrome	5		III
Thrombotic thrombocytopenic purpura	3		I
Neurological disorders	42 (29.2)	119	
Autoimmune encephalitis	22		I
Acute myelitis	6		Not classified
Acute disseminated encephalomyelitis	8		II
Guillain-Barré syndrome	6		I
Poisoning	12 (8.3)	24	
Snake envenomation/mushroom poisoning/drug intoxication	1/2/9		III/II/III
Rheumatologic and immune disorders	6 (4.2)	12	
ANCA-associated vasculitis	1		I
Systemic lupus erythematosus	5		II
Others	3 (2.1)	3	Not classified

“Not classified” indicates diagnoses not listed in the ASFA guidelines. For sub-items, session counts are included in the group totals.

ASFA, American Society for Apheresis (2019 guidelines); ANCA, antineutrophil cytoplasmic antibody.

**Table 3:** TPE-related complications (per session, *N* = 414).

Complication	n (%)
Hypotension	8 (1.9)
Nausea and vomiting	8 (1.9)
Hypothermia	10 (2.4)
Urticaria	8 (1.9)
Hypocalcemia	6 (1.4)
Filter or circuit clotting	30 (7.2)

All complications resolved after prompt management; no treatment-related death occurred.

### 3.2 Outcomes by diagnostic group

The clinical characteristics and outcomes of the different diagnostic groups are compared in Table 4. The incidence of MODS was highest in the sepsis group (89.5%) and lowest in the neurological disorder group (14.3%) (*P* < 0.001). The PRISM III score was highest in the renal disease group (median, 21) and lowest in the poisoning group (median, 12) (*P* = 0.022). Treatment interventions also differed among groups: vasopressor use was most frequent in the sepsis group (57.9%, *P* = 0.035), and the time to TPE initiation was shortest in the poisoning group (median, 7 h) and longest in the sepsis group (median, 72 h) (*P* < 0.001). Mortality was highest in the liver disease group (60.7%) and lowest in the neurological disorder (16.7%) and poisoning (25.0%) groups (*P* = 0.012). The ‘‘others’’ group (*n* = 3) was not included in the intergroup comparison because of the small sample size; one of these 3 patients died, yielding a total of 53 deaths (Table 1).

**Table 4:** Clinical characteristics and outcomes by diagnostic group.

Variable	Sepsis (*n* = 19)	Neurological (*n* = 42)	Liver (*n* = 28)	Hematologic (*n* = 24)	Renal (*n* = 10)	Poisoning (*n* = 12)	Rheumatologic (*n* = 6)	*P* value
Age (months)	48 (9, 132)	83 (36, 120)	30 (12, 108)	48 (36, 93)	72 (31, 144)	78 (36, 144)	132 (72, 150)	0.207
Body weight (kg)	14.0 (10.0, 35.0)	24.0 (15.0, 32.0)	11.5 (10.0, 24.7)	17.0 (14.0, 24.1)	22.2 (12.0, 36.0)	21.5 (13.1, 48.7)	27.5 (19.2, 41.0)	0.192
MODS, *n* (%)	17 (89.5)	6 (14.3)	17 (60.7)	12 (50.0)	6 (60.0)	3 (25.0)	2 (33.3)	<0.001
Mechanical ventilation, *n* (%)	15 (78.9)	18 (42.9)	16 (57.1)	12 (50.0)	3 (30.0)	4 (33.3)	3 (50.0)	0.136
Vasopressor use, *n* (%)	11 (57.9)	5 (11.9)	8 (28.6)	9 (37.5)	3 (30.0)	4 (33.3)	1 (16.7)	0.035
PRISM III score	19 (16, 27)	17 (13, 20)	19 (14, 20)	19 (16, 23)	21 (17, 26)	12 (12, 23)	15 (12, 16)	0.022
TPE sessions	2 (1, 3)	3 (3, 3)	2 (2, 6)	3 (1, 3.7)	2.5 (1.7, 4.2)	1 (1, 2.7)	2 (1, 3)	0.018
Time to TPE initiation (hours)	72.0 (48.0, 144.0)	48.0 (24.0, 141.0)	41.5 (6.0, 100.0)	62.5 (24.0, 177.0)	29.0 (24.0, 72.0)	7.0 (3.2, 14.2)	17.5 (5.2, 19.7)	0.001
PICU length of stay (days)	16.0 (9.0, 32.0)	12.5 (9.0, 31.5)	12.0 (7.0, 31.0)	10.5 (7.0, 24.0)	17.0 (8.7, 24.2)	5.0 (4.2, 8.7)	6.5 (4.5, 26.7)	0.045
Total hospital stay (days)	22.0 (9.0, 57.0)	28.5 (21.0, 40.0)	15.0 (7.0, 31.0)	16.5 (9.7, 35.7)	22.5 (16.5, 32.2)	6.0 (4.2, 10.7)	14.5 (4.5, 16.2)	<0.001
PICU mortality, *n* (%)	10 (52.6)	7 (16.7)	17 (60.7)	10 (41.7)	3 (30.0)	3 (25.0)	2 (33.3)	0.012

Data are presented as median (interquartile range) or *n* (%).

The “others’ group (*n* = 3) was not included in the intergroup comparison because of the small sample size; one of these 3 patients died, yielding a total of 53 deaths among the 144 patients (Table 1).

MODS, multiple organ dysfunction syndrome; PRISM III, Pediatric Risk of Mortality III; TPE, therapeutic plasma exchange; PICU, pediatric intensive care unit.

### 3.3 Comparison between survivors and non-survivors

As shown in Table 5, non-survivors had significantly higher rates of MODS (86.8% vs. 19.8%), mechanical ventilation requirement (83.0% vs. 30.8%), and vasopressor use (67.9% vs. 6.6%) than survivors (all *P* < 0.001). Regarding TPE-related variables, the median number of TPE sessions was significantly lower in non-survivors than in survivors (2 vs. 3 sessions; *P* = 0.022), and the median time to TPE initiation was longer in non-survivors than in survivors (72 vs. 38 h), although this difference did not reach statistical significance (*P* = 0.063).

**Table 5:** Comparison between survivors and non-survivors.

Variable	Survivors (*n* = 91)	Non-survivors (*n* = 53)	*P* value
Male sex, *n* (%)	50 (54.9)	33 (62.3)	0.391
Age (months)	72 (36, 120)	47 (14, 114)	0.121
Body weight (kg)	18.3 (13.0, 32.0)	17.0 (10.0, 26.0)	0.077
MODS, *n* (%)	18 (19.8)	46 (86.8)	<0.001
Mechanical ventilation, *n* (%)	28 (30.8)	44 (83.0)	<0.001
Duration of mechanical ventilation (days)	0.0 (0.0, 5.0)	7.5 (2.2, 14.0)	<0.001
Vasopressor use, *n* (%)	6 (6.6)	36 (67.9)	<0.001
Duration of vasopressor support (days)	0.0 (0.0, 0.0)	2.0 (0.0, 5.0)	<0.001
TPE sessions	3 (2, 3)	2 (1, 3)	0.022
PRISM III score	16.0 (13.0, 20.0)	19.5 (17.0, 26.0)	<0.001
Concomitant CRRT, *n* (%)	30 (33.0)	43 (81.1)	<0.001
Time to TPE initiation (hours)	38.0 (18.0, 96.0)	72.0 (24.0, 184.0)	0.063
PICU length of stay (days)	12.0 (7.0, 25.0)	10.5 (5.0, 30.0)	0.362
Total hospital stay (days)	24.0 (15.0, 38.0)	13.0 (5.2, 31.0)	0.001

Data are presented as median (interquartile range) or *n* (%).

MODS, multiple organ dysfunction syndrome; PRISM III, Pediatric Risk of Mortality III; TPE, therapeutic plasma exchange; CRRT, continuous renal replacement therapy; PICU, pediatric intensive care unit.

### 3.4 Multivariate analysis of prognostic factors

Multivariate logistic regression analysis (Table 6) identified vasopressor use (OR = 6.551, 95% CI: 1.310-32.768; *P* = 0.022) and MODS (OR = 5.609, 95% CI: 1.189-26.453; *P* = 0.029) as independent risk factors for mortality. Mechanical ventilation, PRISM III score, concomitant CRRT, and the duration of organ support were not independently associated with death. The number of TPE sessions and the time to TPE initiation were also entered into the model, but neither was independently associated with mortality (Table 6).

**Table 6:** Multivariate logistic regression analysis of factors associated with mortality.

Variable	B	SE	*P* value	OR	95% CI lower	95% CI upper
Vasopressor use	1.880	0.821	0.022	6.551	1.310	32.768
Duration of vasopressor support	0.108	0.088	0.221	1.114	0.937	1.323
Mechanical ventilation	0.722	0.746	0.333	2.058	0.477	8.877
Duration of mechanical ventilation	−0.049	0.032	0.133	0.952	0.894	1.105
PRISM III score	0.049	0.065	0.453	1.050	0.924	1.194
MODS	1.724	0.791	0.029	5.609	1.189	26.453
CRRT	−0.097	0.709	0.891	0.908	0.261	3.849
Number of TPE sessions	0.002	0.114	0.988	1.002	0.801	1.252
Time to TPE initiation	0.001	0.001	0.352	1.001	0.999	1.004

TPE, therapeutic plasma exchange; MODS, multiple organ dysfunction syndrome; PRISM III, Pediatric Risk of Mortality III; CRRT, continuous renal replacement therapy; OR, odds ratio; CI, confidence interval.

## 4 Discussion

In this retrospective analysis of 144 critically ill children who underwent 414 TPE sessions in a PICU, we delineated the clinical application profile and prognostic pattern of pediatric TPE. Neurological disorders (29.2%), liver diseases (19.4%), and hematologic disorders (16.7%) constituted the principal indication groups, in agreement with pediatric series from other centers (10–14). Hematologic diseases such as hemophagocytic lymphohistiocytosis (HLH) and infection-related diseases such as sepsis accounted for a considerable proportion, reflecting the distinctive disease spectrum of critically ill children. Notably, 57.6% of patients were treated for ASFA category III indications, indicating that the evidence base supporting TPE in most of these children remains weak — a situation closely linked to the scarcity of high-quality randomized controlled trials (RCTs) in pediatric apheresis (15, 16).

Regarding safety, the overall complication rate in our cohort was 16.9% per session (70/414), and filter or circuit clotting was the most frequent event (7.2%), consistent with previously reported pediatric data (6, 7, 11, 17, 18). The relatively high proportion of clotting events may be related to insufficiently individualized anticoagulation regimens. Importantly, all complications resolved after timely intervention without compromising completion of the planned treatment course, and no secondary fatal events occurred, underscoring the procedural safety of TPE when performed by an experienced team (6, 17).

We observed an overall PICU mortality of 36.8%, which is comparable to the 28.4% reported in the systematic review and meta-analysis by Panda et al. (19) and the 39% reported by Ekinci et al. (14) in large cohorts of critically ill children; the high mortality likely reflects the greater severity of underlying disease and the limited organ reserve of this PICU population rather than the procedure itself. Stratified analysis revealed marked heterogeneity in outcomes across diagnostic groups: mortality reached 60.7% in the liver disease group, whereas it was considerably lower in the neurological disorder (16.7%) and poisoning (25.0%) groups (*P* = 0.012), indicating that outcomes after TPE vary substantially among diseases, a pattern more likely attributable to the severity and natural history of the underlying disease than to the procedure itself. In pediatric acute liver failure, TPE has been reported to improve native-liver survival and to serve as a bridge to transplantation or spontaneous recovery (20–26); nevertheless, our liver disease subgroup had the worst prognosis, suggesting that children with advanced hepatic failure and MODS may present beyond the window of reversible injury, and that earlier intervention and combined artificial liver support strategies warrant exploration (24–26). In contrast, the favorable outcomes in neurological disorders align with accumulating evidence supporting TPE in autoimmune encephalitis, acute disseminated encephalomyelitis, and Guillain-Barré syndrome (16, 27–30), while the low mortality in the poisoning group may be attributable to the very early TPE initiation in this subgroup (median, 7 h) (31–33).

Multivariate logistic regression identified MODS (OR = 5.609) and vasopressor use (OR = 6.551) as independent predictors of mortality, consistent with the findings of Sık and Demirbuga et al. (11). Both variables are markers of disease severity rather than modifiable procedural factors. Thrombocytopenia-associated multiple organ failure (TAMOF) represents a thrombotic microangiopathy phenotype of sepsis-induced MODS in children, in which ADAMTS-13 deficiency and ultra-large von Willebrand factor multimers drive microvascular thrombosis (34, 35); TPE may reverse this process by replenishing ADAMTS-13, removing prothrombotic and inflammatory mediators, and restoring a functional plasma milieu (15, 35–37). Several pediatric studies have reported that TPE can improve hemodynamics, reduce vasopressor requirements, and reverse organ dysfunction in sepsis-associated MODS (36, 37), although the Surviving Sepsis Campaign pediatric guidelines still consider the evidence insufficient to support a routine recommendation for blood purification in sepsis (38).

Concerning the timing of TPE, the median time to initiation was longer in non-survivors than in survivors (72 vs. 38 h), although the difference did not reach statistical significance (*P* = 0.063). Early TPE is biologically plausible: in septic shock, early initiation has been shown to improve hemodynamics and vascular barrier function in a prospective pilot study (39), and the concept of reversing “plasma failure” before irreversible organ damage has gained support (40). Whether children who already have MODS would benefit from more aggressive and earlier TPE to improve prognosis requires validation in prospective studies. In the meantime, our findings suggest that a rapid assessment system — for example, dynamic monitoring of the PRISM III score (9) — may help identify candidates for treatment before full-blown organ failure develops.

We did not compare mortality across ASFA categories. The ASFA category reflects the strength of evidence supporting TPE for a given disease rather than the severity of illness, and more than half of our patients (57.6%) were treated for category III indications, leaving the numbers in categories I and II too small for a meaningful statistical comparison. The significant difference in mortality across diagnostic groups (Table 4) suggests that the outcome of children receiving TPE is driven mainly by the underlying disease and its severity rather than by the ASFA classification.

Concomitant CRRT was significantly more frequent among non-survivors (81.1% vs. 33.0%), again reflecting greater illness severity rather than a harmful effect of combined therapy. The combined use of TPE and CRRT is technically feasible in children, including infants, and has been successfully applied in pediatric acute liver failure and severe sepsis or septic shock (25, 26, 41, 42); a recent European consensus statement emphasizes standardized, multidisciplinary delivery of extracorporeal blood purification in the PICU (43).

For renal diseases, TPE remains a mainstay for atypical hemolytic uremic syndrome associated with complement factor abnormalities and an established first-line therapy for thrombotic thrombocytopenic purpura (44, 45). In rheumatologic and immune-mediated diseases, plasma exchange has shown benefit in severe ANCA-associated vasculitis in a large RCT (46) and in childhood vasculitis series (47), while for HLH and macrophage activation syndrome, TPE has been reported to reduce ferritin levels and stabilize critically ill children as a bridge to definitive immunochemotherapy, although the evidence is largely observational (48–50).

Several limitations of this study should be acknowledged. First, as a single-center retrospective analysis with a modest sample size (*n* = 144) and uneven disease distribution, some subgroup analyses may have been underpowered. Second, treatment parameters (e.g., plasma exchange volume and anticoagulation regimens) relied on physician experience and lacked standardization, which may affect the comparability of results. Third, no long-term follow-up data were recorded for certain disease categories, such as neurological disorders, precluding assessment of remote outcomes including neurocognitive sequelae. Fourth, this study included only children who underwent TPE; children with potential indications who did not receive the procedure during the same period were not captured, so the selection process cannot be fully characterized. Fifth, without a control group of children with comparable diagnoses and illness severity (e.g., matched by PRISM III score) who did not undergo TPE, the efficacy of TPE cannot be evaluated, and our conclusions are therefore limited to the safety and clinical characteristics of the procedure. Finally, residual confounding cannot be excluded given the observational design.

Despite these limitations, our study provides a practical reference for pediatric TPE. Indications should be selected strictly according to the ASFA guidelines, with category I and II diseases prioritized; for category III diseases, individualized protocols should be developed through multidisciplinary collaboration, and local evidence should be accumulated. Because MODS and vasopressor use are independent adverse prognostic factors, organ function support and hemodynamic stabilization deserve particular attention during TPE in critically ill children.

## 5 Conclusions

TPE is generally safe in critically ill children when performed in an experienced PICU, with no treatment-related death in this 144-case cohort. MODS and vasopressor requirement independently predict mortality, whereas TPE-related variables do not. Indications should be evaluated strictly according to ASFA categories, and organ support together with hemodynamic stability should be emphasized during treatment. Whether earlier TPE initiation improves survival in children with MODS merits prospective, multicenter investigation. Given the absence of a control group, no conclusion regarding the efficacy of TPE can be drawn from this study; our findings pertain to the safety and clinical profile of the procedure.

## Data Availability

The original contributions presented in the study are included in the article/Supplementary Material, further inquiries can be directed to the corresponding author.

## References

[1] Wong EC Balogun RA. Therapeutic apheresis in pediatrics: technique adjustments, indications and nonindications, a plasma exchange focus. J Clin Apher. (2012) 27(3):132–7. 10.1002/jca.21224 22467438

[2] Kim YA Sloan SR. Pediatric therapeutic apheresis. Pediatr Clin North Am. (2013) 60(6):1569–80. 10.1016/j.pcl.2013.08.006 24237988

[3] Hunt EA Jain NG Somers MJ. Apheresis therapy in children: an overview of key technical aspects and a review of experience in pediatric renal disease. J Clin Apher. (2013) 28(1):36–47. 10.1002/jca.21260 23420594

[4] Padmanabhan A Connelly-Smith L Aqui N Balogun RA Klingel R Meyer E. Guidelines on the use of therapeutic apheresis in clinical practice—evidence-based approach from the writing committee of the American society for apheresis: the eighth special issue, 2019. J Clin Apher. (2019) 34(3):171–354. 10.1002/jca.21705 31180581

[5] Connelly-Smith L Alquist CR Aqui NA Hofmann JC Klingel R Onwuemene OA. Guidelines on the use of therapeutic apheresis in clinical practice—evidence-based approach from the writing committee of the American society for apheresis: the ninth special issue. J Clin Apher. (2023) 38(2):77–278. 10.1002/jca.22043 37017433

[6] Taylan C Schaaf A Dorn C Schmitt CP Loos S Kanzelmeyer N. Safety of therapeutic apheresis in children and adolescents. Front Pediatr. (2022) 10:850819. 10.3389/fped.2022.850819 35498796 PMC9039165

[7] Michon B Moghrabi A Winikoff R Barrette S Bernstein ML Champagne J. Complications of apheresis in children. Transfusion. (2007) 47(10):1837–42. 10.1111/j.1537-2995.2007.01405.x 17880609

[8] Goldstein B Giroir B Randolph A. International consensus conference on pediatric sepsis. International pediatric sepsis consensus conference: definitions for sepsis and organ dysfunction in pediatrics. Pediatr Crit Care Med. (2005) 6(1):2–8. 10.1097/01.PCC.0000149131.72248.E6 15636651

[9] Pollack MM Patel KM Ruttimann UE. PRISM III: an updated pediatric risk of mortality score. Crit Care Med. (1996) 24(5):743–52. 10.1097/00003246-199605000-00004 8706448

[10] Özsoylu S Dursun A Çelik B. Therapeutic plasma exchange in pediatric intensive care unit: a single-center experience. Indian J Crit Care Med. (2021) 25(10):1189–92. 10.5005/jp-journals-10071-23985 PMC8645823 34916754

[11] Sık G Demirbuga A Annayev A Akcay A Çıtak A Öztürk G. Therapeutic plasma exchange in pediatric intensive care: indications, results and complications. Ther Apher Dial. (2020) 24(2):221–9. 10.1111/1744-9987.13474 31922326

[12] Dalkiran T Mercan M Ipek S Güllü UU Kandur Y Acipayam C. Therapeutic plasma exchange in pediatric patients: results from a single center. J Pediatr Intensive Care. (2024) 13(3):282–5. 10.1055/s-0041-1742252 39629152 PMC11379518

[13] Bustos B R Hickmann O L Cruces R P Díaz F. Therapeutic plasma exchange in critically ill children: experience of the pediatric intensive care unit of two centers in Chile. Transfus Apher Sci. (2021) 60(5):103181. 10.1016/j.transci.2021.103181 34238709

[14] Ekinci F Yildizdas D Horoz OO Yontem A Acar IH Karadamar M. Therapeutic plasma exchange in critically ill children: 18-year experience of a tertiary care paediatric intensive care unit. Aust Crit Care. (2024) 37(4):592–9. 10.1016/j.aucc.2023.12.006 38331694

[15] Fortenberry JD Nguyen T Grunwell JR Aneja RK Wheeler D Hall M. Therapeutic plasma exchange in children with thrombocytopenia-associated multiple organ failure: the thrombocytopenia-associated multiple organ failure network prospective observational study. Crit Care Med. (2019) 47(3):e173–81. 10.1097/CCM.0000000000003559 30531184 PMC9084562

[16] Eyre M Hacohen Y Barton C Hemingway C Lim M. Therapeutic plasma exchange in paediatric neurology: a critical review and proposed treatment algorithm. Dev Med Child Neurol. (2018) 60(8):765–79. 10.1111/dmcn.13925 29856068

[17] Chowdhry M Mutjhukamaravel PJ Agrawal S Gajulapali SP Thakur UK. Assessing safety and efficacy of therapeutic plasma exchange in pediatric patients: a single-center experience. Asian J Transfus Sci. (2022) 16(1):61–6. 10.4103/ajts.AJTS_25_20 36199402 PMC9528540

[18] Eichinger K Breu M Renken M Siegert S Hilz E Glatter S. Complications of therapeutic plasma exchange in pediatric neuroimmune disorders. Children (Basel). (2025) 12(11):1457. 10.3390/children12111457 41300575 PMC12651235

[19] Panda K Dash DP Panda PK Dash M Saboth PK Pati GK. Role of therapeutic plasma exchange on survival in pediatric acute liver failure and acute-on-chronic liver failure: a systematic review and meta-analysis. Indian J Gastroenterol. (2025) 44(5):618–33. 10.1007/s12664-025-01742-2 40035795

[20] Alexander EC Deep A. Therapeutic plasma exchange in children with acute liver failure (ALF): is it time for incorporation into the ALF armamentarium? Pediatr Nephrol. (2022) 37(8):1775–88. 10.1007/s00467-021-05289-0 34647173 PMC9239959

[21] Biswas T Lal BB Sood V Ashritha A Maheshwari A Bajpai M. Therapeutic plasma exchange provides native liver survival benefit in children with acute liver failure: a propensity score-matched analysis. J Clin Apher. (2024) 39(3):e22130. 10.1002/jca.22130 38873972

[22] Chowdhry M Sharma A Agrawal S Vohra R Kumar K Goyal N. Efficacy of therapeutic plasma exchange in pediatric cases of acute liver failure as an extracorporeal liver support system. Transfus Apher Sci. (2023) 62(6):103835. 10.1016/j.transci.2023.103835 37996345

[23] Lim H Kang Y Park S Koh H. Effectiveness of high-volume therapeutic plasma exchange for acute and acute-on-chronic liver failure in Korean pediatric patients. Pediatr Gastroenterol Hepatol Nutr. (2022) 25(6):481–9. 10.5223/pghn.2022.25.6.481 36451692 PMC9679303

[24] Gao Q Chen J Zhao C Li J Song A Zhang Z. Combination of plasma exchange and adsorption versus plasma exchange in pediatric acute liver failure: a multicenter cohort study. J Pediatr Gastroenterol Nutr. (2023) 76(6):710–5. 10.1097/MPG.0000000000003759 36913704

[25] Chongsrisawat V Samransamruajkit R Trepatchayakorn S Chaijitraruch N Chanakul A Kongkiattikul L. Therapeutic plasma exchange with continuous renal replacement therapy for pediatric acute liver failure: a case series from Thailand. Indian J Crit Care Med. (2021) 25(7):812–6. 10.5005/jp-journals-10071-23896 34316178 PMC8286369

[26] Jackson C Carlin K Blondet N Jordan I Yalon L Healey PJ. Continuous renal replacement therapy and therapeutic plasma exchange in pediatric liver failure. Eur J Pediatr. (2024) 183(8):3289–97. 10.1007/s00431-024-05587-3 38717620

[27] Ipe TS Meyer EK Sanford KW Joshi SK Wong ECC Raval JS. Use of therapeutic plasma exchange for pediatric neurological diseases. J Clin Apher. (2021) 36(1):161–76. 10.1002/jca.21850 33063869

[28] Yıldırım M Bektaş Ö Botan E Şahin S Gurbanov A Teber S. Therapeutic plasma exchange in clinical pediatric neurology practice: experience from a tertiary referral hospital. Clin Neurol Neurosurg. (2021) 207:106823. 10.1016/j.clineuro.2021.106823 34304066

[29] Agarwal S Keller JR Nunneley CE Muscal E Braun MC Srivaths P. Therapeutic plasma exchange use in pediatric neurologic disorders at a tertiary care center: a 10-year review. J Child Neurol. (2018) 33(2):140–5. 10.1177/0883073817749368 29334853

[30] Borras-Novell C García Rey E Perez Baena LF Jordan Garcia I Catella Cahiz D Cambra F. Therapeutic plasma exchange in acute disseminated encephalomyelitis in children. J Clin Apher. (2015) 30(6):335–9. 10.1002/jca.21388 26332469

[31] Sun X Chen X Lu J Tao Y Zhang L Dong L. Extracorporeal treatment in children with acute severe poisoning. Medicine (Baltimore). (2019) 98(47):e18086. 10.1097/MD.0000000000018086 31764843 PMC6882618

[32] Özkale M Özkale Y. The role of therapeutic plasma exchange in the treatment of childhood intoxication: a single-center experience. Pediatr Crit Care Med. (2020) 21(11):e988–95. 10.1097/PCC.0000000000002462 32701752

[33] Yildirim C Bayraktaroğlu Z Gunay N Bozkurt S Köse A Yilmaz M. The use of therapeutic plasmapheresis in the treatment of poisoned and snake bite victims: an academic emergency department's Experiences. J Clin Apher. (2006) 21(4):219–23. 10.1002/jca.20094 16619226

[34] Nguyen TC Carcillo JA. Bench-to-bedside review: thrombocytopenia-associated multiple organ failure—a newly appreciated syndrome in the critically ill. Crit Care. (2006) 10(6):235. 10.1186/cc5064 17096864 PMC1794442

[35] Nguyen TC Han YY Kiss JE Hall MW Hassett AC Jaffe R. Intensive plasma exchange increases ADAMTS-13 activity and reverses organ dysfunction in children with thrombocytopenia-associated multiple organ failure. Crit Care Med. (2008) 36(10):2878–87. 10.1097/CCM.0b013e318186aa49 18828196 PMC2772176

[36] Sevketoglu E Yildizdas D Horoz OO Kihtir HS Kendirli T Bayraktar S. Use of therapeutic plasma exchange in children with thrombocytopenia-associated multiple organ failure in the turkish TAMOF network. Pediatr Crit Care Med. (2014) 15(8):e354–9. 10.1097/PCC.0000000000000227 25068251 PMC5287151

[37] Kawai Y Cornell TT Cooley EG Beckman CN Baldridge PK Mottes TA. Therapeutic plasma exchange may improve hemodynamics and organ failure among children with sepsis-induced multiple organ dysfunction syndrome receiving extracorporeal life support. Pediatr Crit Care Med. (2015) 16(4):366–74. 10.1097/PCC.0000000000000351 25599148 PMC4424057

[38] Weiss SL Peters MJ Alhazzani W Agus MSD Flori HR Inwald DP. Surviving sepsis campaign international guidelines for the management of septic shock and sepsis-associated organ dysfunction in children. Intensive Care Med. (2020) 46(Suppl 1):10–67. 10.1007/s00134-019-05878-6 32030529 PMC7095013

[39] Knaup H Stahl K Schmidt BMW Idowu TO Busch M Wiesner O. Early therapeutic plasma exchange in septic shock: a prospective open-label nonrandomized pilot study focusing on safety, hemodynamics, vascular barrier function, and biologic markers. Crit Care. (2018) 22(1):117. 10.1186/s13054-018-2220-9 30373638 PMC6206942

[40] Foglia MJ Raval JS Hofmann JC Carcillo JA. Therapeutic plasma exchange to reverse plasma failure in multiple organ dysfunction syndrome. J Clin Apher. (2024) 39(5):e22147. 10.1002/jca.22147 39420549

[41] Aygün F Varol F Durak C Talip Petmezci M Kacar A Dursun H. Evaluation of continuous renal replacement therapy and therapeutic plasma exchange in severe sepsis or septic shock in critically ill children. Medicina (Kaunas). (2019) 55(7):350. 10.3390/medicina55070350 PMC6680968 31284692

[42] Ide K Muguruma T Shinohara M Toida C Enomoto Y Matsumoto S. Continuous veno-venous hemodiafiltration and plasma exchange in infantile acute liver failure. Pediatr Crit Care Med. (2015) 16(8):e268–74. 10.1097/PCC.0000000000000511 26237654

[43] Bottari G Buccione E Bayrakci B Briassoulis G Carter MJ Demirkol D. Extracorporeal blood purification in European pediatric intensive care units: a consensus statement. JAMA Netw Open. (2025) 8(2):e2457657. 10.1001/jamanetworkopen.2024.57657 39899300

[44] Hans R Sharma RR Marwaha N Suri D Kumar R Gupta A. Efficacy and safety of therapeutic plasma exchange by using apheresis devices in pediatric atypical hemolytic uremic syndrome patients. J Clin Apher. (2016) 31(4):381–7. 10.1002/jca.21412 26212115

[45] Carter CE Benador NM. Therapeutic plasma exchange for the treatment of pediatric renal diseases in 2013. Pediatr Nephrol. (2014) 29(1):35–50. 10.1007/s00467-013-2479-7 23812351

[46] Walsh M Merkel PA Peh C-A Szpirt WM Puéchal X Fujimoto S. Plasma exchange and glucocorticoids in severe ANCA-associated vasculitis. N Engl J Med. (2020) 382(7):622–31. 10.1056/NEJMoa1803537 32053298 PMC7325726

[47] Wright E Dillon MJ Tullus K. Childhood vasculitis and plasma exchange. Eur J Pediatr. (2006) 166(2):145–51. 10.1007/s00431-006-0212-2 16915376

[48] Bosnak M Erdogan S Aktekin EH Bay A. Therapeutic plasma exchange in primary hemophagocytic lymphohistiocytosis: reports of two cases and a review of the literature. Transfus Apher Sci. (2016) 55(3):353–6. 10.1016/j.transci.2016.09.015 27743708

[49] Demirkol D Yildizdas D Bayrakci B Karapinar B Kendirli T Koroglu TF. Hyperferritinemia in the critically ill child with secondary hemophagocytic lymphohistiocytosis/sepsis/multiple organ dysfunction syndrome/macrophage activation syndrome: what is the treatment? Crit Care. (2012) 16(2):R52. 10.1186/cc11256 22715953 PMC3681377

[50] Canna SW Marsh RA. Pediatric hemophagocytic lymphohistiocytosis. Blood. (2020) 135(16):1332–43. 10.1182/blood.2019000936 32107531 PMC8212354

